# Freezing the Electronic Wave: Uncovering the Atomistic Structure of Charge Density Waves in 1D Metallic MOFs

**DOI:** 10.1021/acscentsci.6c00952

**Published:** 2026-06-12

**Authors:** Matthew J. Cliffe

**Affiliations:** Department of Materials Science and Metallurgy, 2152 University of Cambridge, 27 Charles Babbage Rd, Cambridge CB3 0FS, U.K.

## Abstract

Advanced crystallographic determination of the structures of porous metallic metal–organic frameworks uncovers strong evidence of quantum electronic behavior.

## Graphical abstract

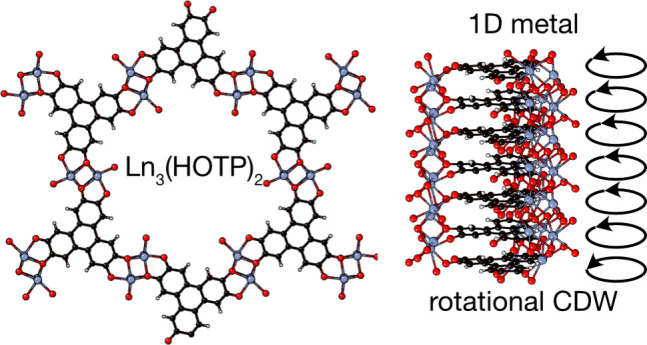

Metal–organic frameworks (MOFs) give chemists the tools to be atomic scale architects, designing and synthesizing porous materials with atomic precision.^1^ Chemists have used this to develop MOFs with specific pore structures, creating remarkable adsorbents capable of capturing water from desert air, detecting and removing PFAS “forever chemicals,” and encapsulating proteins for use in therapeutics. These applications have focused on the extraordinary negative space of MOFs, but the framework itself can have remarkable physical properties.

One of the most exciting developments in MOF chemistry is the discovery that the electrons in MOFs are not confined to individual metal clusters or linkers: highly electrically conductive and even metallic MOFs can be made. As this conductivity depends on the pathways of the mobile electrons through the framework, the MOF geometry will dictate the reciprocal-space electronic band structure. The electronic band structure is fundamental to the electronic properties of a material, determining whether it is a semiconductor, metal, or even superconductor. Control over the geometry of conductive MOFs could therefore be used to design and realize targeted quantum electronic phases. Thus far, however, there are no clear examples of quantum electronic properties in MOFs.

The electronic band structure is fundamental to the electronic properties of a material, determining whether it is a semiconductor, metal, or even superconductor. Control over the geometry of conductive MOFs could therefore be used to design and realize targeted quantum electronic phases.

In a recent issue of *ACS Central Science*, Mircea Dincă, Julius Oppenheim, and co-workers provide the strongest evidence yet of an electronic phase transition in a MOF.^2^ They use advanced crystallographic analysis, including high-pressure measurements, to uncover the atomistic structure of a charge density wave (CDW) in the family of rare-earth MOFs [Ln­(NO_3_)_1–*x*_]_3_(HOTP)_2_, Ln = La, Ce, Pr, Nd, and Sm, HOTP = hexahydroxytriphenylene, which have quasi-1D metallicity due to the 1D stacks of partially oxidized HOTP ligands (Figure 1).

**Figure 1:**
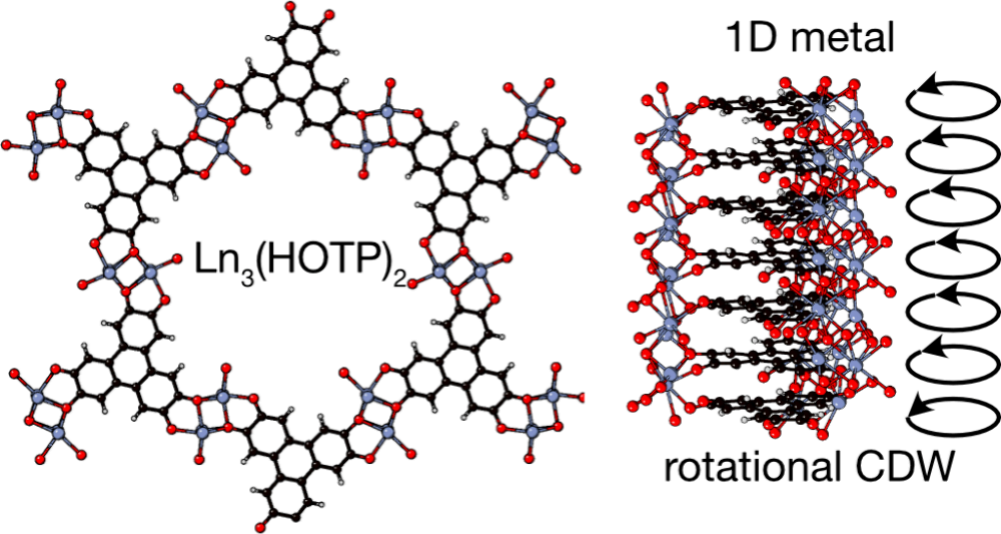
LnHOTP has a structure comprising 2D sheets with a kagome arrangement of Ln ions connected by three-coordinate HOTP ligands. The Ln ions form infinite lanthanide oxide chains, causing the HOTP ligands to be closely stacked. The close proximity generates 1D metallicity, which undergoes a charge density wave distortion comprising rotations of the ligands (shown here for an arbitrary periodicity).

They use advanced crystallographic analysis, including high-pressure measurements, to uncover the atomistic structure of a charge density wave (CDW) in the family of rare-earth MOFs [Ln­(NO3)_1–*x*_]_3_(HOTP)_2_, Ln = La, Ce, Pr, Nd, and Sm, HOTP = hexahydroxytriphenylene, which have quasi-1D metallicity due to the 1D stacks of partially oxidized HOTP ligands.

Charge density waves are quantum states that emerge from the fundamental instability of the 1D metallic state to distortions. The most famous example is the coupling of a 1D metallic band to vibrations, which can freeze in place and produce a gap in the band structure, generating a semiconductor (Peierls distortion, Figure 2a). This concept is intuitive to chemists, both in its analogy to the Jahn–Teller distortion in electronically degenerate molecules and in the explanation of why undoped polyacetylene forms alternating single and double bonds.^3^ Metal–insulator transitions in 1D metals have a rich range of behavior, with such transitions also caused by the correlation between electrons (the Mott transition) or coupling to magnetic order, and are closely connected to other complex electronic phases, notably superconductivity.^4^

**Figure 2:**
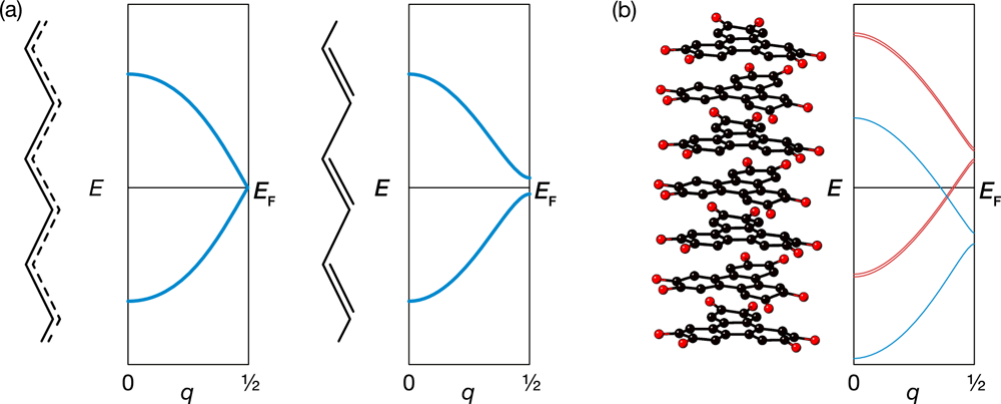
(a) The electronic band structure of polyacetylene with equal bonds is metallic, which cuts the Fermi energy (*E*_F_) at the zone boundary *q* = 1/2, shown schematically. The 1D metal is unstable to a Peierls type charge density wave distortion, which doubles the unit cell and hence splits the band at the zone boundary, generating a gap. (b) The LnHOTP family has three metallic bands due to the 1D stack of HOTP^*x*–^ ligands, which cut the Fermi energy at a range of periodicities, depending on the bandwidth and band filling, giving a range of CDW periodicities.

In this paper, Ryu et al. build on their previous discovery of the LnHOTP family and their observation of a low-temperature transition. By carrying out a detailed X-ray diffraction study that combines low-temperature and high-pressure crystallography, they find convincing evidence that this transition has CDW character, together with a wide range of subtle but important structural variations.

The diversity of structure is apparent in the striking differences in the X-ray diffuse scattering found in these materials, implying a wide range of local orderings. The authors show, using detailed Monte Carlo modeling, that this arises from different local orderings of the rare earth ion with respect to the ligand, which depends not only on the identity of the lanthanide but also on the synthesis protocol.

More complexity emerges on cooling below around 350 K, where a phase transition occurs in most of the materials. This phase transition was detected by the emergence of new sharp Bragg peaks in the X-ray diffraction patterns, at positions distinct from the diffuse scattering. These new peaks indicate that the structures now have a longer periodicity along the stacking direction, *c*, than in the high-temperature structures. This periodicity of the “modulation” again varied between samples, including doubling of the unit cell (**q** = 0.5 c*), tripling (**q** = 0.33 c*), and even incommensurate modulations without a rational relationship to the underlying MOF structure (**q** = 0.24 c*). By solving the structure of the material and the modulation, the authors were able to uncover that this phase transition is driven by the rotation of the planar HOTP ligand. The periodic variation in rotation breaks the symmetry of the metallic bands arising from the HOTP stacking and produces an electronic gap (Figure 2b). Interestingly, these materials remain highly conductive, and density functional theory calculations suggest this is because only one of the two metallic bands is broken by this distortion.

The authors provide more striking evidence of the CDW character in high-pressure studies exploring how increasing orbital overlap changes the electronic structure and hence the nature of the CDW. They show that the wavelength of the CDW phases decreases with applied pressure for two lanthanides as the bandwidth increases, but stays fixed at a 3-fold supercell for La. This “lock in” is a strong indicator of an energetic stabilization of a CDW phase and so is the best evidence yet of such a phase in a porous MOF.

This work establishes that LnHOTP is a family of materials with great potential. It asks a number of intriguing questions about the nature of the ground state of these materials and the origin of the structural and CDW differences observed between different lanthanides and synthesis conditions. It also suggests that tuning of LnHOTP, whether through higher pressure, ligand functionalization, or charge doping, might realize new and interesting electronic phases, as has been found in dense metal–organic CDW materials such as Cu­(dicyanoquinonediimine)_2_.^5^

The work of Ryu et al. will inspire the search for CDWs and quantum electronic states in MOFs beyond LnHOTP. Higher-dimensional structures are particularly exciting: are there 2D MOF conductors with electronic phases analogous to the superconducting AV_3_Sb_5_ kagome metals^6^ or 3D MOF metals with extended orbital molecules similar to those found in the vanadate spinels?^7^ This work has opened the door to a world of electronic phases for MOF chemists and condensed matter physicists to explore—who knows what they will find.
